# Do journals instruct authors to address sex and gender in psychological science?

**DOI:** 10.1186/s41073-020-00100-4

**Published:** 2020-10-22

**Authors:** Courtenay Cavanaugh, Yara Abu Hussein

**Affiliations:** Rutgers University, Camden, NJ USA

**Keywords:** Journal instructions to authors; sex, Gender, Equity, Reproducibility, Research design and reporting

## Abstract

**Background:**

Sex and gender influence individuals’ psychology, but are often overlooked in psychological science. The sex and gender equity in research (SAGER) guidelines provide instruction for addressing sex and gender within five sections of a manuscript (i.e., title/abstract, introduction, methods, results, and discussion) (Heidari et al., Res Integr Peer Rev 1:1-9, 2016).

**Methods:**

We examined whether the 89 journals published by the American Psychological Association provide explicit instruction for authors to address sex and gender within these five sections. Both authors reviewed the journal instructions to authors for the words “sex,” and “gender,” and noted explicit instruction pertaining to these five sections.

**Results:**

Only 8 journals (9.0%) instructed authors to address sex/gender within the abstract, introduction, and/or methods sections. No journals instructed authors to address sex and gender in the results or discussion sections.

**Conclusion:**

These journals could increase sex/gender equity and improve the reproducibility of psychological science by instructing authors to follow the SAGER guidelines.

Sex and gender affect individuals’ psychology throughout the lifespan. Thus, sex and gender must be carefully considered in all aspects of psychological science including the reporting of research in journal publications. Poor attention to sex and gender in scientific studies is bad for science as it affects the reproducibility of science and can obscure sex and gender differences [1]. It also results in real harm, particularly for women, as 8 of 10 drugs pulled from the market in a 3 year period were more harmful to women [2]. A recent review of the top two psychopathology journals from 2010 to 2018 found that 58 studies did not report sex/gender in the title/abstract, or participant section [3] and another review of sex and gender differences in substance use revealed that many studies did not conduct gender-specific analyses, nor do they have adequate representation of women in clinical trials [4].

The Sex and Gender Equity in Research (SAGER) guidelines guide authors in addressing sex and gender within five sections of an article including the title/abstract, introduction, methods, results, and discussion [5]. For example, specifying in the title and abstract if the study only applied to one sex or gender. Other recommendations are to report in the introduction if sex and gender differences are to be expected and to conduct sex- and gender-based analyses [5]. Journal editors have been called to implement these recommendations [1, 5, 6]. This study examined whether journals published by the American Psychological Association (APA) instruct authors explicitly to address sex and/or gender within the five sections of the manuscripts they submit for publication.

## Methods

Ninety-one APA journals were identified [7]. These journals pertain to different subjects in psychology (e.g., basic/experimental, clinical, health psychology, and social psychology). Between December 2019–January 2020, both authors went to each journals homepage (e.g. https://www.apa.org/pubs/journals/ort/), and searched for the terms “sex” and “gender” on each journals “manuscript submission” tab (e.g., https://www.apa.org/pubs/journals/ort/?tab=4) or “submit” tab which contained instructions to authors for preparing their manuscripts. Any time the terms “sex” and/or “gender” were found, the authors determined whether the related statement directly instructed authors to address sex and gender issues within the title/abstract, introduction, methods, results or discussion sections of the manuscript. Two of the 91 journals (i.e., Clinician’s Research Digest: Adult Populations and Clinician’s Research Digest: Child and Adolescent Populations) did not have a manuscript submission tab and were therefore not included in this report. Thus, this report pertains to 89 APA journals.

## Results

### Instructions for authors to address sex and gender within the five sections of the manuscript

Eight of the 89 (9.0%) APA journals had explicit instructions for authors to address sex or gender within the abstract, introduction, and/or methods sections of their manuscripts. Specifically, four journals instructed authors to describe the gender of the study sample within the abstract, one journal instructed authors to address gender in the introduction, and five journals instructed authors to describe the gender of the study sample in the methods section of the manuscript. No APA journals instructed authors to address sex and gender in the results or discussion sections of the manuscript. No journals provided specific instructions for authors to specify the sex/gender of study participants in the study title and abstract if the study only pertained to one sex or gender.

## Conclusion

Our findings may not be consistent with what psychological journals not published by the APA instruct authors to do. Nevertheless, this study is important as findings reveal that APA journals are failing to provide authors with explicit instructions for addressing sex and gender in different sections of their manuscripts. Only 9.0% of the 89 journals examined had any instruction for authors to address sex and gender within specific sections of their manuscripts. The fact that no journals instructed authors to conduct sex- and gender-specific analyses is particularly concerning since not doing so may both obscure sex and gender differences in psychological science and hinder the reproducibility of psychological science [1, 8] or what is known at the replication crisis in psychology [9]. We must consider how many findings in psychological science are assumed to generalize to people of different sexes and genders even when the research design may not have allowed such conclusions to be drawn. To improve psychological science, we call upon APA journals to do the following: 1) add and expand instructions for authors to address sex and gender in all sections of their manuscripts [5], 2) provide all journal editors and reviewers with the SAGER guidelines [5], and 3) have manuscripts evaluated upon their adherence to these guidelines.

## Data Availability

All the data and materials for this study may be obtained by contacting the first author of this paper at cocavana@camden.rutgers.edu
